# Iconic versus working memory metacognition to evaluate the richness of perception: a registered report

**DOI:** 10.1098/rsos.231805

**Published:** 2025-11-12

**Authors:** Nicolás Alejandro Comay, Guillermo Solovey, Pablo Barttfeld

**Affiliations:** ^1^ Cognitive Science Group. Instituto de Investigaciones Psicológicas (IIPsi, CONICET-UNC), Facultad de Psicología, Universidad Nacional de Córdoba, Córdoba, Argentina; ^2^ Laboratorio de Neurociencia, Universidad Torcuato Di Tella, Buenos Aires, Argentina; ^3^ Escuela de Negocios, Universidad Torcuato Di Tella, Buenos Aires, Argentina; ^4^ Instituto de Cálculo, Facultad de Ciencias Exactas y Naturales, UBA-CONICET, Buenos Aires, Argentina

**Keywords:** consciousness, metacognition, iconic memory, working memory, confidence

## Abstract

The high capacity of human iconic memory (IM) has been taken as evidence that visual experience is rich and detailed, as introspection suggests. Opponents to this view argue instead that this impression is illusory, with conscious access being mostly limited to what we can attend to. To provide evidence of either view, in this registered report we compared metacognitive sensitivity levels between IM and working memory (WM) representations. The rationale was that, if pre-attentive IM information is as consciously accessible as attention-bounded WM information, metacognitive sensitivity should be comparable across the two memory systems. Replicating classic findings, our results showed that IM capacity exceeded WM capacity. Nevertheless, and despite matched performance, metacognitive sensitivity was higher in WM. We further examined whether reduced metacognition in IM could be explained by inflation—the tendency to overestimate perceptual richness—by comparing confidence levels across the two memory conditions. Pre-registered analyses showed no evidence of inflation, as IM was associated with lower confidence. Our findings suggest that IM supports identification with less consciously accessible information than WM, challenging rich-view interpretations of conscious perception.

## 1. Introduction

A vast number of stimuli are constantly processed by our visual system. Most of them only elicit transient sensory responses, while others reach consciousness, subsequently affecting our behaviour. Introspectively, our visual experience of the world seems rich and detailed, fostering the notion that the conscious perception of the world is very informative and we are aware of most of its content. Indeed, partial report paradigms have shown that, at early stages of visual perception, we can report a surprisingly high amount of details: when humans see an intricate array of stimuli, a retrospective, immediately presented cue helps subjects to retrieve a much higher amount of information than when no cue is given [1–4], a classic result suggesting the existence of a high capacity, *iconic* memory (IM) lasting a few hundred milliseconds after stimulus presentation. However, this evidence contrasts with reliable findings that consistently reveal the conscious perception of stimuli to be much more limited than our introspection might suggest [5–8]. For instance, in inattentional blindness paradigms subjects focus on a primary task and fail to notice unexpected stimuli, and, similarly, in change blindness paradigms subjects fail to notice how natural scenes continuously change [8].

These conflicting findings—limited conscious access coupled with an introspective feeling of richness regarding visual experience—have led to two opposite views [4,6,9]: while some authors have argued that conscious perception is attention-dependant and limited to what people can report [5]—and therefore the introspective feeling of richness is deceptive—others support the view that conscious perception is rich and detailed, and limited attentional resources precludes this information from being available for report [10,11].

The complexity of this issue is further compounded when taking into account multiple factors that influence conscious perception. For instance, in tasks that induce high perceptual load (i.e. multiple features of the stimuli need to be integrated), stimuli may be filtered out in early stages of processing. Conversely, in low perceptual load situations, information is processed but remains unavailable for report owing to failures in memory, not in perception [12,13]. In addition to this, research on visual perception points out that only summaries of unattended peripheral stimuli are represented in the brain [6,14]. This perspective suggests that the combined effects of losing peripheral information and the demands of specific tasks could explain the observed shortfalls in perceptual tasks [14]. Therefore, high perceptual load and peripheral limitations point out that some information may be lost during the early phases of sensory processing. This leads to the view that the apparently rich phenomenology is because of some form of inflation: we interpret the sensory information as if it were richer than it actually is [12,15,16]. The inflation account is mainly supported by experiments that found more liberal decision and confidence criteria when comparing attended and unattended conditions. In other words, humans tend to report more frequently perceiving stimuli in the unattended peripheral field, often with elevated confidence levels, even when task performance is equivalent ([17–21]; although see [22,23]).

Despite the quality of the information present at sensory stages, the key question is whether this information is consciously perceived or not [12], i.e. whether IM information is conscious. Vandenbroucke *et al*. [4] addressed this issue by comparing metacognitive sensitivity for working versus sensory memory (i.e. fragile and IM) on a change detection task and on an identification task. As they found similar levels of metacognition between conditions, they concluded that sensory memory representations must be as explicit as working memory (WM) representations, providing evidence for the ‘rich and detailed’ view.

However, it has been argued that, as Vandenbroucke *et al*. [4] experiments used visual cues signalling a specific target item spatial position before the test display onset, that metacognitive ability cannot be generalized to the entire visual field. In other words, the same metacognitive sensitivity cannot be assumed for all items in the absence of a cue and its concomitant attendant process [24]. In summary, there is still controversy about whether IM representations are conscious.

To address this issue, the present work builds on the Vandenbroucke *et al*. [4] study comparing metacognition for IM and WM representations, following the assumption that metacognitive ability indexes consciousness [25]. As different questioning procedures can influence the amount of content that is qualified as consciously perceived [9], to overcome limitations of the Vandenbroucke *et al*. [4] study [24] our experimental protocol did not involve the use of visual spatial cues. Specifically, we presented a circular array of letters for 250 ms. Participants had to report the position of a specific letter and report their confidence on having made a correct choice. Critically, participants did not know which letter we would inquire about—as no spatial cue was present—and therefore the entire stimuli array had to be taken into consideration to solve the task. As a consequence, in this design the assessment of metacognitive ability extends to the whole visual field instead of being restricted to a particular cued item as in Vandenbroucke *et al*. [4] and usually done. In the IM condition subjects had to report a specific letter after 250 ms of stimuli offset. In this context, the decision is executed as in classic perceptual decision making, where it is assumed that sensory information guides the decisions [26]. In the WM condition, to elicit greater involvement of WM, subjects had to report a specific stimuli position after 8000 ms of stimuli offset. We conducted an identification task (similar to experiment 2 in [4]) rather than a change detection task (similar to experiment 1 in [4]) since change detection tasks can induce differences in the decision criterion between memory conditions [4]. Critically, this might influence metacognitive measures such as the area under a type 2 receiver operating characteristic (AUROC-2), which was used in the present study (see [27] and §3.2.1). Moreover, we used letters instead of differently oriented bars (Vandenbroucke *et al.* stimuli) to prevent that the number of stimuli increases items’ similarity.

We next present our research questions and hypothesis, summarized in table 1 together with the statistical tests to prove the hypothesis and interpretations of the outcomes obtained. Moreover, figure 4 also depicts a diagram for the possible outcomes and their interpretation.

**Table 1.** Summary of the pre-registered research questions, hypotheses, sampling plan, analysis plan and interpretations under the present study.

research question	hypothesis	sampling plan	analysis plan	interpretation given to different outcomes
**RQ1:** do we have similar subjective knowledge of IM and WM representations?	**H1:** the AUROC2 on the WM condition will be higher than on the IM condition	*n* = 101. statistical power (SP) = 80%	paired samples *t*‐test to address whether $\theta_{AUROC2}\neq 0$. Equivalence test to address whether $\theta_{AUROC2}$ is between the equivalence bounds	if $\theta_{AUROC2}>0$ and $\theta_{AUROC2}$ is not between the equivalence bounds, we will interpret this result as evidence against the ‘rich and detailed’ view, as the subjective knowledge about IM representations is not as explicit as WM information. However, in the case that we find a significant difference in the *t*‐test but, at the same time, $\theta_{AUROC2}$ is between the equivalence bounds, we will state that although a significant difference was found, this difference is not sufficiently large to matter (i.e. it is smaller than the smallest effect size of interest), and we will conclude that the conditions are statistically equivalent (the same equivalence interpretation will hold if the *t*‐test is not significant). This means that the information in the IM representation is as explicit as the information in the WM representation, thus supporting the rich and detailed view. We will also interpret support for this view if metacognition is significantly higher for IM (i.e. $\theta_{AUROC2}<0$, note that the interpretation favouring this view holds regardless of the result of the equivalence test). Importantly, these interpretations favouring the ‘rich and detailed’ view will be conditioned on the results of RQ3 (see below). Finally, if the *t*‐test is not significant and $\theta_{AUROC2}$ is not between the equivalence bounds, we will state that we did not find conclusive evidence to answer the RQ1 (and in consequence RQ2), as conditions are not statistically different but at the same time not statistically equivalent
**RQ2:** is an inflation-like mechanism at play at the IM level?	**H2:** confidence will be higher on the IM condition	*n* = 101. SP > 80%	ordered mixed regression model to test whether $\beta_{IM}$ is significantly higher than zero	if $\beta_{IM}>0$, then we will conclude that an inflation-related mechanism can explain the phenomenological feeling of perceptual richness. In the case that this result is accompanied by similar metacognitive levels between conditions or higher IM metacognition in RQ1, we will not interpret this as inflation, as typically inflation is related to diminished metacognitive ability. If $\beta_{IM}\approx 0$ (i.e. not statistically different from zero) or $\beta_{IM}<0$ we will interpret this outcome as no evidence for an inflation-like mechanism being at play
**RQ3:** do we have higher item capacity on the IM on identification tasks?	**H3:** item capacity will be higher on IM when compared with WM	*n* = 101. SP > 99%	paired samples *t*‐test to address whether $\theta_{IC}<0$	if $\theta_{IC}<0$, we will conclude that the higher capacity on the IM is preserved despite the absence of a visual spatial cue. If not, we will conclude that the additional attentional processing that the visual spatial cue provides is a necessary condition for the higher capacity of the IM and, importantly, regarding RQ1 we will not interpret similar metacognitive levels between conditions as evidence favouring the ‘rich and detailed’ view (as in this scenario conscious perception appears to be limited to what we can attend). We will interpret that the subjective knowledge about the two types of memories is similar, but it does not imply richness in the whole (unattended) visual field. An alternative interpretation for this scenario of equal metacognitive levels and equal item capacity is that the memory manipulation was simply ineffective, thus rendering trivial results at the metacognitive level. if $\theta_{IC}\approx 0$ coupled with greater metacognitive sensitivity for IM (i.e. $\theta_{AUROC2}<0$ and $\theta_{AUROC2}$ is not between the equivalence bounds) are found, we will interpret that the subjective knowledge about sensory information is greater than for WM information, but we will not state that this favours the rich and detailed view as conscious perception seems to be limited to what we can attend to (as stated previously). We will interpret this result as a difference in between the two memory types (i.e. the subjective knowledge for IM is higher than the one for WM), but not implying richness extended to unattended parts of the visual field. if, unexpectedly, $\theta_{IC}>0$, the same stated interpretations for $\theta_{IC}\approx 0$ will hold (with the exception of the ‘ineffective manipulation’ alternative, as in this case there is a difference between conditions)
**experimental control:** is performance different between IM and WM?	**H4:** performance will be equal between conditions	*n* = 101. SP > 99%	equivalence test to address whether $\theta_{PERF}$ is between the equivalence bounds	if $\theta_{PERF}$ is between the equivalence bounds, we will conclude that our staircase procedure worked properly. If the equivalence test is not significant, we will conclude that performance between conditions are not statistically equivalent. In this case, we will state that this is a limitation of our study, and that the differences —or absence of them— in research questions 1 to 3 should be interpreted with care as conditions are not fully comparable regarding task performance

## 2. Research questions

### 2.1. Research question 1 (RQ1): do we have similar levels of subjective knowledge of iconic and working memory representations?

With the aim to address whether the information of the IM representation is consciously perceived, we introduce a paradigm similar to the one in Vandenbroucke *et al*. [4]: we compare metacognitive sensitivity for IM and WM representations but, critically, we exclude the use of visual spatial cues, addressing prior criticisms [24]. If the information of the IM is consciously perceived, metacognitive ability should be comparable across conditions. On the contrary, *our hypothesis (H1a) was that metacognitive sensitivity would be higher in the WM condition*. We also evaluated whether this difference, if present, was sufficiently large to matter using a statistical equivalence test, that can also provide evidence for the claim that the two conditions are equivalent ([28], see §3). Our *hypothesis (H1b) was that the difference between conditions would be sufficiently large to reject the statistical equivalence hypothesis*.

As task performance has been shown to affect metacognitive measures [27], we controlled for performance on both conditions using a staircase procedure (see §3).

### 2.2. Research question 2 (RQ2): is an inflation-like mechanism at play at the iconic memory level?

The role of attention has been proposed to be critical for the presence of the inflation effect, as stated previously. In our experimental protocol, it is expected that higher attention levels will be present in the WM condition when compared with the IM condition [15]. Indeed, traditionally attention has been regarded as the ‘gatekeeper’ of WM [29–32]. This led to the view that only a small portion of attended visual input reaches WM—although see Kaunitz *et al*. [33] and Matthews *et al*. [34] for evidence for long-lasting memory capacity with robust metacognitive sensitivity even for incidentally seen distractor stimuli. By contrast, IM has been thought of as a pre-attentive memory with almost unlimited capacity [35]. In addition to this, in the proposed experimental paradigm: (i) potentially fewer items will be presented in the WM condition (owing to the staircase procedure), meaning that more attentional resources are directed to each individual item; and (ii) even in the case of comparable item capacity, items in the WM condition must be maintained and focused for a longer period. This difference in attention levels might lead to noisier percepts in the IM condition compared with the WM condition [19,20], therefore causing a rise in confidence levels owing to variance misperception (i.e. humans do not take into account the increased variability in the sensory information in one condition compared with another [36]). Crucially, variance misperception has been proposed as a computational mechanism for inflation [21] and it is not an intrinsically spatial phenomenon [36]. Consequently, although we are not manipulating spatial attention directly as previous inflation-related studies, higher confidence levels on the IM condition would provide evidence for an inflation-like mechanism. In line with this possibility, Vandenbroucke *et al*. [4] found more liberal change detection criteria in the sensory memory conditions when compared with the WM condition, an effect interpreted by the authors to be analogous to the inflation effect.

Therefore, we tested whether an inflation-like effect is present, but at the confidence level (as in [18,21]) instead of at the decision level, as our task does not involve stimuli detection but identification. Our *hypothesis (H2) was that confidence will be higher on the IM condition*. We controlled for two possible confounds: number of items and accuracy (see §3).

### 2.3. Research question 3 (RQ3): do we have higher item capacity on the iconic memory on identification tasks?

It has been argued that IM is a high capacity type of memory as we can recover a high amount of details when a cue is given shortly after stimuli presentation. Therefore, our *hypothesis (H3) was that larger item capacity (i.e. the mean number of items shown) would be found in the IM condition when compared with the WM condition*. It has to be acknowledged, however, that this effect can be smaller than in change detection tasks, as identification tasks can be more attention-dependent [4].

## 3. Methods

The entire experimental protocol and analyses reported here were pre-registered in a stage 1 Registered Report procedure, available at: https://doi.org/10.17605/OSF.IO/AGJ45. No deviations from the pre-registered procedures were made.

### 3.1. Experimental protocol

#### 3.1.1. Participants

Participants were undergraduate or graduate university students between 18 and 40 years old. Participants with a history of neurological damage, psychiatric conditions and/or chronic psychoactive drugs consumption were not included. Colourblind participants were not included. Participants received 5 USD for taking part in the study.

A total of 141 participants (63.83% female; mean age = 23.21 years, s.d. = 4.14) completed the study. Of these, 40 were excluded according to pre-registered criteria (see stage 1 protocol and §3.5.1). Specifically, participants were excluded if they had fewer than 180 valid trials in any condition after removing trials with (i) response times (RT) below 150 ms in either the decision or confidence judgement, (ii) no response within 4000 ms for decisions or 3000 ms for confidence judgements, or (iii) fewer than four items.

Among the excluded subjects, on average 0.52 trials (s.d. = 0.99) were discarded in the WM condition and 0.62 (s.d. = 1.10) in the IM condition owing to slow RT (decision RT > 4 s or confidence RT > 3 s). This difference was not significant (*t*_39_ = –0.73, *p* = 0.47). With respect to fast RT (decision or confidence RT < 0.15 s), on average 14.9 trials (s.d. = 15.91) were discarded in the WM condition and 23.27 (s.d. = 22.17) in the IM condition. This difference was significant (*t*_39_ = –4.98, *p* < 0.001) with 8.37 trials more, on average, being discarded in the IM condition. Finally, 10.5 (s.d. = 17.32) trials on average were discarded in the WM condition and 6.77 (s.d. = 10.08) in the IM condition owing to insufficient number of items presented (i.e.: less than four items criterion). The difference between conditions was not significant (*t*_39_ = 1.51, *p* = 0.139).

To ensure that the excluded subjects do not affect the results, we ran the pre-registered analyses again but without excluding any subjects and found the same results for the three main research questions and the performance control. We report these results in the electronic supplementary material. The final sample consisted of a total of 101 final participants (69.31% females, mean age = 23.22, s.d. age = 4.07), which matched the pre-registered sample size for this study.

#### 3.1.2. Stimuli

Participants were presented with an array of letters for a duration of 250 ms. These letters were displayed randomly on *N* equispaced positions in a circular array (where *N* represents the number of letters on each trial). The circular array rotated randomly in every trial, meaning that letter positions changed across trials. The radius of the circular array had 8.81° of visual angle. Participants sat at a distance of 70 cm to the screen. Letter size was 1.92° of visual angle horizontally and 2.87° of visual angle vertically. On each trial, one specific letter was randomly picked to be the target from the following set: {B, C, D, F, G, H, J, K, L, M, N, P, Q, R, S, T, V}. In order to reduce the possibility of crowding effects, letters had different colours [37,38]. These colours were intercalated between letters (i.e. no neighbouring letters had the same colour). Letters were coloured with five different colours in the case of 5, 9 and 13 items presented in the stimuli array, otherwise letters were coloured with four different colours; this guaranteed that no adjacent letters had the same colour. Colours were sampled from the Commission Internationale de l’Eclairage (CIE) L*a*b* space. We fixed luminance (L*) at 70 and sampled four and five equidistant points in the {a*; b*} plane that were also equidistant from the grey point (a* = 0; b* = 0), with a distance between adjacent points of 149.2975 units to make colours very different from each other (and equally different regardless of whether four or five colours were presented). Adjacent letters were coloured with the adjacent points in colour space, meaning that the difference in colours between neighbouring letters will be fixed at the mentioned 149.2975 units in CIE L*a*b* space.

The following colours (rounded up to four decimals) were used when four colours were present in the stimuli array (each value of the vector representing L*a*b* values respectively): [70, 105.5692, 0], [70, 0, 105.5692], [70, –105.5692, 0] and [70, 0, –105.5692]. The following colours were used when five colours were present in the stimuli array: [70, 127, 0], [70, 39.2452, 120.7842], [70, –102.7452, 74.64873], [70, –102.7452, –74.6487] and [70, 39.2452, –120.7842]. Note that the precision of the colours presented was restricted to the capacity of the monitor, that may not represent all decimals.

#### 3.1.3. Equipment

The experiment ran in Matlab using the Psychophysics Toolbox [39]. Stimuli were displayed on a LG 24GL600F LED monitor of 23.6 inches with a 144 Hz refresh rate.

An Eyelink 1000 plus eye-tracker (SR Research) using a fixed-head configuration through a chinrest was used to ensure participants were fixating on the fixation point at the beginning of each trial. We considered a central fixation if the gaze was located within a centred square with 2° of visual angle side length.

#### 3.1.4. Procedure

Participants performed two experimental sessions on different days. In the first experimental session, the experimenter explained the procedure and gave the informed consent and the information sheet to the participants. After reading both documents, if the participant accepted and signed the informed consent, the experiment began. Instructions were presented on the screen. The experimenter asked if the participant had any question(s). Then the eye-tracking set-up, calibration and validation was performed. After that, the experimenter left the participant alone and proceeded to monitor the eyetracker. The task consisted of four blocks, with the eye-tracker being recalibrated and revalidated between blocks. A message appeared on the screen to the participant at the conclusion of each block, indicating that the block has ended and requesting the participant to contact the experimenter for eye-tracker recalibration. Participants were allowed to rest for a couple of minutes between blocks. The procedure was repeated for the second session.

**Figure 1.**
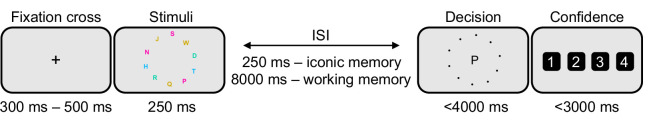
Iconic and working memory metacognition task. A fixation cross appeared on the centre of the screen with a presentation time sampled from a uniform distribution between 300 and 500 ms. Stimuli were different letters. An inter-stimulus-interval of 250 ms (iconic memory condition) or 8000 ms (working memory condition) separated the offset of the stimuli with the onset of the decision question. Participants had to decide where the target item appeared on screen before 4000 ms. Critically, as no cue is given before the decision question, participants must necessarily attempt to retain awareness of all items presented up to the moment of decision. After that, they reported their confidence on having made the correct decision before 3000 ms.

The experimental task is displayed in figure 1. A fixation cross appeared on the centre of the screen. The presentation time of this fixation cross was sampled from a uniform distribution between 300 and 500 ms. After this period, the trial started only if the subject fixates on the cross. The trial did not begin until the subject fixed their gaze. The letter array was shown for 250 ms. After that, a question stating ‘Where was the letter X?’—where ‘X’ stands for a randomly selected letter from the letter set—was presented on the centre of the screen. This question was presented only for the first seven practice trials; after that, only the target letter was presented. Depending on the experimental condition (IM or WM), this question appeared with an inter-stimulus-interval (ISI) of 250 ms (IM condition), or 8000 ms (WM condition). The potential positions were indicated with dots appearing at the onset of the decision question, and participants were required to click on the location they believed the target item occupied. On each trial the target item was sampled randomly from the set of letters presented in the array. Participants were asked to make their decision before 4000 ms, otherwise a message for 10 s appeared on the screen warning the participant that she/he took too much time to respond. After that the next trial began. After making the decision, participants had to report their confidence on a 4-point scale by clicking in any of the four confidence buttons. In this confidence-reporting stage, a question in all trials was placed centred on the top of the screen stating ‘How sure are you?’. Participants had to report their confidence before 3000 ms, otherwise a message for 10 s appeared on the screen warning the participant that she/he took too much time to respond. After that the next trial began. All of these quotes were in Spanish (see figure 1), i.e. ‘¿*Dónde estaba la letra X*?’ for the decision question; ‘*¿Qué tan seguro/a estás*?’ for the confidence question.

After reporting their confidence, the next trial automatically began with an inter-trial-interval uniformly sampled between 850 ms and 1150 ms. Each experimental session had 10 practise trials and 130 trials per memory condition (making a total of 270 trials per session, which leaves 400 valid trials for analysis in total—see §3.5.1). Pauses for rest and recalibration of the eyetracker were placed after experimental trials numbered 65, 130 and 195. At the end of each experimental session a blank response box was provided to the participants to—if they wanted—give feedback about their feelings and strategies used throughout the experiment and also to give us suggestions (these responses were not analysed).

#### 3.1.5. Experimental manipulations

Each trial was randomly an IM (250 ms ISI) or WM (8000 ms ISI) trial. Two independent (i.e. one per condition) staircase procedures were used to achieve similar performance levels for both conditions. After one correct trial an item was added to the next trial stimuli array, whereas after one incorrect trial an item was subtracted from the next trial stimuli array. If the participant did not make a decision within 4000 ms then that given trial was not considered for the staircase, and the same number of stimuli was used for the next trial of the same memory condition. These staircases had an upper bound on 16 items. Our pilot data (see the electronic supplementary material) suggested that this limit was unlikely to be reached: only 6 of 1163 valid trials were 16-item trials. Moreover, these trials were all incorrect responses, meaning that if this limit was reached, it was unlikely to be maintained.

This procedure guarantees that, after stabilization (approximately 20–30 trials), 50% of trials will yield correct responses.

### 3.2. Variables

The independent variable was a categorical one: the memory condition. Each trial randomly was an IM or a WM trial. Both conditions were presented to each participant, thus constituting a within-subjects design.

Dependent variables were the proportion of correct responses on each condition, the mean number of items shown on each condition (i.e. item capacity), the AUROC-2 curve (i.e. metacognitive sensitivity, see [27]) on each condition, and the mean confidence level on each condition.

#### 3.2.1. Computation of the area under a type 2 receiver operating characteristic

The AUROC-2 measure considers all possible confidence criteria that can split confidence levels into high and low. In order to obtain the AUROC-2, we computed the proportion of ‘type 2’ hits (high confidence in a correct response) and ‘type 2’ false alarms (high confidence on an incorrect response) for each division of the data done by these confidence criteria on each memory condition. In the context of our experiment, we considered a correct response choosing the correct location of the target letter and an incorrect response otherwise. For constructing the receiver operating characteristic (ROC) curve, the inverse cumulative type 2 hits rate is plotted on the *y*-axis, and the inverse cumulative type 2 false alarm rate is plotted on the *x*-axis. The line that crosses those points is the ROC curve, and the area under this curve is the AUROC-2. Importantly, this measure is particularly suited for measuring metacognitive sensitivity in the present experimental design as it can accommodate the multi-alternative nature of the task [40].

### 3.3. Sample size and power analysis

As stated above, associated with the mentioned variables we have three main research questions (RQ): do we have similar subjective knowledge of IM and WM representations? (RQ1); is an inflation-like mechanism at play at the IM level? (RQ2); and compared with WM, do we have higher capacity on IM in identification tasks? (RQ3). We will now describe the rationale behind the sample size chosen, stating the statistical power achieved for the three RQ.

To calculate a sample size that leads to sufficient power (≧80%) to answer RQ1, we first defined our smallest effect size of interest (SESOI [28]);—i.e. the smallest effect size that is sufficiently large to matter. Since we leverage on a conceptually similar previous study [4], we used the results of that work as a basis for our effect size calculation. When one is basing the SESOI in a previous study, one possibility is to use the ‘small-telescopes’ approach [28,41]. This procedure implies that one should set the SESOI as the standardized effect size that the earlier study would have had 33% power to detect. Following this, we used the second experiment in Vandenbroucke *et al*. [4]—conceptually more similar to the one proposed here as it was a kind of identification task—to calculate our SESOI. A post hoc power analysis shows that, with an alpha level of 0.05 and 24 subjects, the standardized effect size that the study would have 33% power to detect is equal to 0.325 (calculated using the G*Power software). Therefore, we performed our sample size calculations with the aforementioned targeted minimum effect size.

As we not only want to provide evidence for the alternative hypothesis (H1a) but also to discern whether the difference is sufficiently large to matter (H1b), we will perform both a *t*‐test and an equivalence test between memory conditions [28]. Our desired type I error rate will be 5%, but since we will perform two statistical tests with the same data we will use a 2.5% type I error rate to correct for multiple comparisons in order to calculate our sample size for RQ1 [42].

Taking all of this into account, we performed two power analysis calculations: one for the *t*‐test and one for the equivalence test. Regarding the former, a sample size of 93 subjects is needed in order to have an 80% power to detect a difference between metacognition in both conditions (sensory versus WM), with a standardized effect size equals 0.325 and a 0.025 probability of type 1 error, as calculated using the G*Power software. Regarding the latter, to achieve 80% power to detect a difference between metacognition in both conditions (sensory versus WM), with equivalence bounds of standardized effect sizes of −0.325 and 0.325 and a 0.025 probability of type 1 error, a sample size of 101 subjects is needed (as calculated using the ‘TOSTER’ package in R). Therefore, we included 101 subjects in our experiment (note that this led to a power of 83.7% for the first *t*‐test, as calculated in a post hoc power analysis using the G*Power software).

The difference between memory conditions regarding metacognitive sensitivity represents our main research question (RQ1), but is not the only research question that we have. Therefore, the above mentioned effect size is not the only effect size at play. We now state how the *n* = 101 impacts the statistical power for RQ2 and RQ3.

In our RQ2, we compare confidence ratings between conditions to evaluate whether an inflation-like mechanism is at play. To control for possible confounds regarding the number of items presented and the accuracy of the response, we employed a mixed ordered regression model to evaluate whether confidence differs between memory conditions (see §3.5). To compute our achieved statistical power, we simulated confidence responses for 101 subjects with 400 trials each across several values of the coefficient for the IM condition. The coefficients for the other predictors were fixed at the values that we obtained on the pilot data (see the electronic supplementary material, table S1). We repeated this process 100 times per IM coefficient, and computed the proportion of these simulated experiments that yielded a statistically significant result regarding the IM coefficient (i.e. the statistical power achieved). We found that with *n* = 101 and a type I error of 0.05 the smallest coefficient that we can detect as being significant with greater than 80% power is 0.035. We simulated 100 experiments with this coefficient value and computed the Cohen’s *d* statistic to be able to compare it with the (absolute) effect size reported by Odegaard *et al*. [18] of 1.76—obtained using the $d=|\frac{t}{\sqrt{n}}|$ formula [43]. We found that this coefficient produces a mean Cohen’s *d* of 0.26 (s.d. = 0.09). In consequence, our sample size allows us to detect with 80% power a much smaller effect than reported in the previous literature, meaning that enough power is achieved to answer RQ2.

Regarding RQ3, item capacity has been found to be significantly larger on IM in both experiments performed in Vandenbroucke *et al*. [4]. With the reported *F* statistics and degrees of freedom (d.f.), we calculated the partial eta-squared with the following formula [43]:


$$n_{p}^{2}=\frac{F\;*\;d.f._{effect}}{F\;*\;d.f._{effect}\;+\;d.f._{error}},$$

which results in a partial eta-squared equal to 0.56 for experiment 1 and a partial eta-squared equal to 0.24 for experiment 2 [4]. We computed the resulting power for detecting the smallest of these effects (i.e. 0.24) with our sample size using the G*Power software. Using an ANOVA that mirrors the *t*‐test that we will actually perform, with 101 subjects, one group, two measurements and a type I error of 0.05, the statistical power obtained is greater than 99% for all the correlations among repeated measures possible (i.e. {−0.999; 0.999}; also note that the nonsphericity correction is fixed to 1 in this analysis).

Finally, as an experimental control, we expected that performance would not be different between memory conditions as we included a staircase procedure. We used the raw equivalence bounds of −0.05 and 0.05 to address if these conditions are statistically equivalent, meaning that a difference with raw effect size in between these predefined boundaries is too small to be of interest. With *n* = 101, an alpha level of 0.05, a standard deviation of the difference between conditions of 0.01 (estimated from the pilot data; see the electronic supplementary material for the pilot data) and the mentioned raw equivalence bounds, our statistical power to detect a significant statistical equivalence is greater than 99%. Assuming that our standard deviation of the difference estimate can be noisy, we tested whether this power holds with a standard deviation of the difference between conditions as high as 0.1 and found that with *n* = 101 the statistical power is still greater than 99%.

### 3.4. Rule for terminating data collection

Data collection concluded upon reaching 101 valid participants as stated by the reported power analysis.

### 3.5. Data analysis procedures

All analyses were performed using the software R.

#### 3.5.1. Rules for data exclusion

The data of a participant was excluded if the participant stated in the box at the end of the experiment that she does not want her data to be included. No participant was excluded owing to this reason.

Practise trials and the first 30 trials of each memory condition—trials prior to the staircases stabilization—were excluded from the analysis. This left 400 valid trials per participant (i.e. 270 trials per session minus 10 practise trials and 60 staircase stabilization trials).

We then excluded trials with (i) RTs shorter than 150 ms both for decision and confidence reports, (ii) no decision before 4000 ms and/or no confidence report before 3000 ms, and (iii) less than four items displayed. If more than 10% of the trials of any condition got excluded owing to these reasons (i.e. any condition ended up with less than 180 trials), the participant’s data was discarded.

If a participant got excluded owing to any of the aforementioned reasons, we replaced this participant with a new one to yield the desired *n* = 101.

#### 3.5.2. Statistical tests

On each experiment we computed, for each participant and on each memory condition, the proportion of correct responses (i.e. performance), the mean number of items shown (i.e. item capacity), the AUROC-2 curve (i.e. metacognitive sensitivity [27]) and the mean confidence level.

The mentioned RQ associated with those variables were mapped to hypothesis, statistical tests and interpretations as described below (see also table 1 and figure 4).

#### 3.5.3. Research question 1 (RQ1): do we have similar levels of subjective knowledge of iconic and working memory representations?

To address this question we evaluated whether metacognitive sensitivity, operationalized as the AUROC-2, is different between memory conditions. With that aim, we computed the AUROC-2 for each participant on each memory condition, and using a dependent samples *t*‐test, we evaluated whether the two conditions are statistically different. We represent this difference with the parameter $\theta_{AUROC2}$, i.e. WM metacognition—IM metacognition. Under the mentioned *t*‐test, the null hypothesis states that $\theta_{AUROC2}$ is equal to zero and the alternative hypothesis states that it is different from zero. Our hypothesis (H1a) was that $\theta_{AUROC2}>0$, as we expected that participants will have greater metacognitive sensitivity on the WM condition. However, as stated, we also tested whether this difference was sufficiently large to matter using an equivalence test. In other words, if we find a difference in between conditions regarding the metacognitive ability, is this difference sufficiently large to matter? Under this test, the null hypothesis states that $\theta_{AUROC2}<\;lower\;equivalence\;bound$ and that $\theta_{AUROC2}>upper\;equivalence\;bound$, where the lower and upper confidence bounds are −0.325 and 0.325, respectively (see §3.3 for the rationale behind these equivalence bounds). On this test, the alternative hypothesis states that $\theta_{AUROC2}$ is between the equivalence bounds. Our hypothesis (H1b) was that there will be no evidence for the alternative hypothesis on the equivalence test, meaning that the two conditions are not statistically equivalent. That is, we expected to find a difference on the *t*‐test favouring the WM condition (i.e. metacognitive ability will be higher on the WM condition) and also that this difference will be sufficiently large to not reject the null hypothesis (i.e. that there is a difference) in the equivalence test.

As two tests will be performed on the same data, we corrected for multiple comparisons by using a type I error rate of 2.5% for these analyses.

#### 3.5.4. Research question 2 (RQ2): is an inflation-like mechanism at play at the iconic memory level?

We tested this question by using an ordered mixed regression model with a probit link function, as in this case confidence is an ordinal variable (subjects’ can only respond in a 1–4 scale). This model assumes that confidence is a continuous latent variable where thresholds are placed to predict the different categories of the outcome (in this case, the confidence levels reported by the subjects). The mean of the latent variable distribution depends on the value of the linear predictor. Specifically, we predicted the confidence on each trial by subject i using: the number of items (nItems) displayed on that particular trial, a binary indicator (accuracy) stating whether the response was correct (1) or incorrect (0) and a binary indicator (IM) of whether the trial was an IM trial (1) or a WM trial (0). As responses are nested within subjects, we added to the latent mean a random intercept by subject. The model was coded in R using the *clmm* function of the *ordinal* package [44] as follows (‘*model 1’*, see §3.5.7 for further model specifications):


confidence  ∼  IM+nItems+accuracy+(1|subject).

Our hypothesis (H2) was that confidence would be higher on the IM condition, a result that would count as evidence favouring the inflation account (see §2.2 and table 1). On the stated model, this means that the IM predictor (represented as $\beta_{IM}$) should be positive and statistically different from zero. Importantly, the model controls the influence that both the number of items and the correctness of the response may have on confidence, thus isolating the effect produced by the IM condition. As suggested by a reviewer, we checked whether the model is robust to correlation between the IM and the nItems predictor (as presumably participants would have higher item capacity in the IM condition, see RQ3 below). We found that the model can deal with high correlation levels (we tested correlations levels from 0.07—pilot data—to 0.77) between the true predictors as 90% or more of the estimated confidence intervals included the true beta values (see Review history for more details).

#### 3.5.5. Research question 3 (RQ3): do we have higher item capacity on the iconic memory on identification tasks?

Our hypothesis (H3) was that the item capacity on the IM condition would be higher than the item capacity on the WM condition. For testing this hypothesis, we computed the mean quantity of items by subject and memory condition. Thus, we obtained two values per subject: one value representing the mean quantity of items in the IM condition, and one value representing the mean quantity of items in the WM condition. Then, using all these values from all subjects, we used a dependent samples *t*‐test to address whether the item capacity differs between conditions. We represented this difference (where the subtraction is WM—IM, as in RQ1) with the parameter $\theta_{IC}$. We expected that $\theta_{IC}$ would be significantly lower than zero. Under the mentioned test, the null hypothesis states that $\theta_{IC}=0$ and the alternative hypothesis is that $\theta_{IC}\neq 0$.

#### 3.5.6. Experimental control: is performance different between iconic and working memory?

To avoid performance confounds we implemented two independent staircases to make the proportion of correct responses on each condition equal to 0.5. Our hypothesis (H4) was that performance between each memory condition will not be different. We mapped this hypothesis into an equivalence test, with raw effect size bounds of {−0.05; 0.05}, as stated in §3.3. In order to do it, we first computed the performance of each subject on each memory condition by taking the proportion of correct responses on that particular subject and condition (similarly to the statistical tests for RQ1 and RQ3). We represent the difference on performance between the two conditions with the parameter $\theta_{PERF}$. Under the equivalence test, the null hypothesis states that $\theta_{PERF}<\;lower\;equivalence\;bound$ and that $\theta_{PERF}>upper\;equivalence\;bound$, where $\theta_{PERF}$ represents the difference between memory conditions. Conversely, the alternative hypothesis states that $\theta_{PERF}$ is between the equivalence bounds. We expected that conditions turn out to be statistically equivalent (i.e. that $\theta_{PERF}$ will be in between the equivalence bounds).

#### 3.5.7. Exploratory analyses

We also conducted two sets of non-pre-registered analyses. One was aimed at exploring whether variability in the number of stimuli presented on each condition could account for the differences obtained in metacognition. The other was implemented to evaluate whether a differential effect of confidence in the IM condition was present between correct and incorrect trials, inline with previous results related to the inflation account [18].

Regarding the first set of exploratory analyses, it has been shown that staircases can artificially inflate metacognition by mixing easy and hard trials, because they make it easier for an observer to distinguish correct and incorrect responses with its confidence judgements since easy trials get high confidence ratings and difficult trials low confidence ratings [45]. In our paradigm, two independent staircases were applied on each condition. Each staircase subtracted one item after an incorrect response and added one item after a correct response by condition (i.e.: 1-up/1-down staircases). Therefore, if for whatever reason this procedure resulted in one condition having a more variable number of items across trials, it would mean that more easy (low number of items) and difficult trials are mixed. This could be problematic because it could create spurious differences between conditions regarding metacognitive levels. We conducted three analyses for controlling this. First, we computed the standard deviation of the number of items presented on each condition and compared them using a paired *t*‐test. Second, we conducted a regression analysis predicting the AUROC-2 values on each condition using the (normalized) standard deviation of the number of items presented on each condition and a binary indicator for the WM condition. Normalization ensured that the beta value for the WM condition reflected the difference in metacognition at the average value of the standard deviation of items found in the data (instead of at zero, which is meaningless as it is impossible in our experimental design owing to the staircases). Finally, we computed the most frequent set size on each memory condition by subject, and computed metacognitive sensitivity by condition using only the trials with that specific set size. We compared the obtained metacognitive sensitivity using a paired samples *t*‐test and an equivalence test, with an alpha level of 0.025 to correct for the multiple comparisons with the same data.

The second set of exploratory analyses we performed aimed to check whether the effect of the IM condition was different between correct and incorrect trials. Indeed, Odegaard *et al*. [18] found that the inflation effect was specifically restricted to incorrect trials, but our pre-registered regression analysis included accuracy as a main effect without any interaction with the IM condition predictor. This precludes the possibility of detecting a differential effect on confidence of the IM condition between correct and incorrect trials. Therefore, we tested a regression model with an interaction term, which was coded in R using the *ordinal* package [44] as follows (‘*model 2*’):


confidence ∼ IM∗accuracy+nItems +(1|subject).

The conventions of the predictors’ names are the same as the ones used in the original analysis. Owing to a reviewer’s suggestion, we also conducted the same regression analysis but with random effects for all the predictors (‘*model 3*’), which is indeed recommended for within subjects designs [46]:


confidence ∼ (1+IM∗accuracy+nItems | subject).

We compared these last two models using the Aikake’s information criterion (AIC) to correct for the extra parameters added for the random effects.

## 4. Results

### 4.1. Results for pre-registered analyses

**Figure 2.**
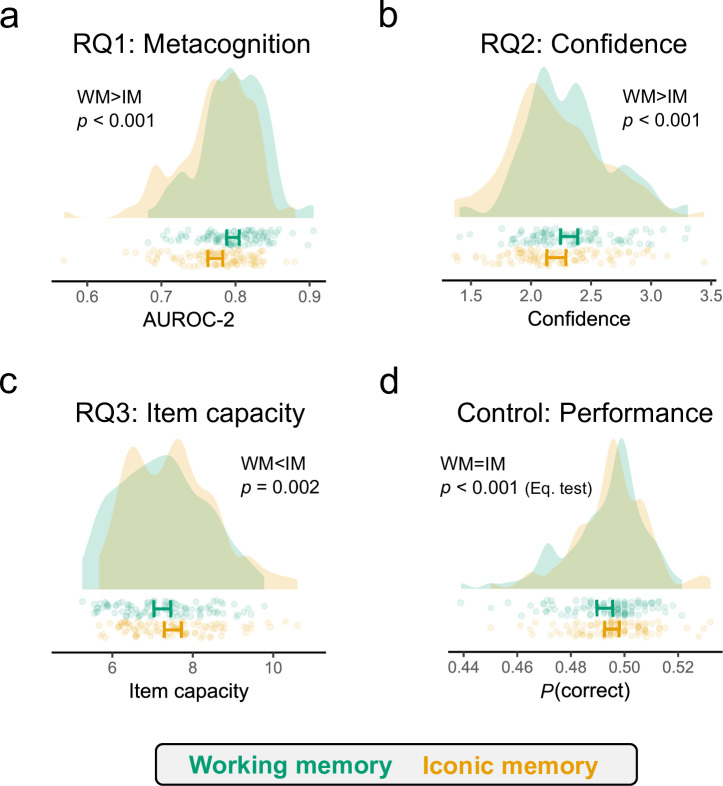
Main results. (a) We found significantly higher metacognitive sensitivity levels in the working memory condition. (b) Lower confidence levels were found in the iconic memory condition. (c) Greater item capacity was found in the iconic memory condition. (d) Equivalent accuracy levels were found in both conditions. In all panels and figures points represent individual participant averages, and error bars represent the 95% confidence interval. Note that, on each panel, the *x*-axis represents different units of measurement and therefore are not comparable between panels.

Regarding our first and most important research question—do participants have similar metacognitive sensitivity levels between memory conditions?—we found that metacognitive ability was significantly higher in WM (*t*_100_ = 4.68, *p* < 0.001, *d* = 0.47; figure 2a). Moreover, this effect was sufficiently large to matter, as the pre-registered equivalence test was not significant (*t*_100_ = 1.41, *p* = 0.92).

Our second research question concerned the presence of an inflation-like effect, where higher confidence ratings should be found in the IM condition. Contrary to this prediction, the ordered regression results suggest that confidence levels were significantly lower in the IM condition ($\beta_{IM}$ = –0.08, s.e. = 0.01, *p* < 0.001; figure 2b). Confidence was also negatively affected by the number of items present ($\beta_{n\;stim}$ = –0.12, s.e. = 0.01, *p* < 0.001), and positively associated with response accuracy ($\beta_{accuracy}$ = 1.28, s.e. = 0.01, *p* < 0.001).

Finally, our third research question evaluated whether participants would present higher item capacity (i.e. a greater set size on average) in the IM condition compared with the WM condition. Indeed, a classic result in the literature is that IM has a higher capacity than WM. Consistently, we found that the mean number of items shown in the IM condition was significantly greater than in the working memory condition (*t*_100_ = –3.22, *p* = 0.002, *d* = 0.32; figure 2c).

Overall, our results support: a positive answer to RQ1, a negative answer to RQ2 and a positive answer for RQ3. To ensure that these results are not confounded by differences in performance, we employed two independent staircase procedures (i.e. one per memory condition) to achieve an accuracy rate of around 0.5 for all participants. Performance was indeed close to this value for both IM (*M* = 0.50; s.d. = 0.01) and WM (*M* = 0.49; s.d. = 0.01) conditions (figure 2d). Furthermore, and as pre-registered, we conducted an equivalence test with raw equivalence bounds of {–0.05; 0.05} and found that both conditions were indeed statistically equivalent (*t*_100_ = 28.17, *p* < 0.001; figure 2d). These results provide evidence that performance was not a variable influencing the reported results.

### 4.2. Exploratory analyses

**Figure 3.**
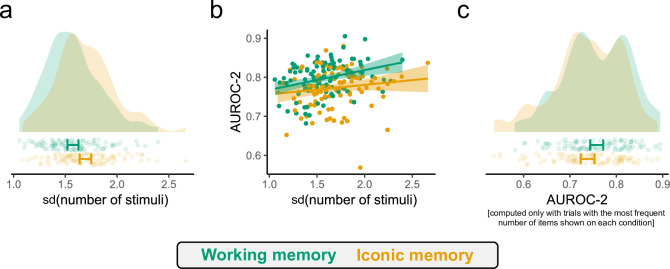
Exploratory controls of the effect of item variability in metacognitive scores. (a) Higher variability in the number of items presented was found in the iconic memory condition. (b) Although a positive trend is presented in both conditions, the variability of the number of items presented did not significantly predict the metacognitive ability of the participants. Lines and shaded regions illustrate a linear fit. (c) Using only the trials with the most frequent number of items presented on each condition we still find higher metacognitive sensitivity in the working memory condition.

To further control for potential confounds in RQ1, we evaluated whether variability in set size had an impact in our results. Previous work has shown that the stimulus variability which the staircases induce can inflate metacognitive metrics, with greater variability leading to higher metacognitive scores [45]. First, we compared the standard deviation of the number of stimuli shown in both conditions and found that the IM had a significantly higher variability with respect to the number of items presented on screen (*t*_100_ = –3.80, *p* < 0.001, *d* = 0.38; figure 3a). This means that, if anything, metacognitive sensitivity levels were overestimated more in the IM condition. Next, using linear regression, we evaluated whether the (normalized) standard deviation of the number of stimuli shown predicted the AUROC-2 levels of the participants. This regression also included as a predictor a binary indicator for the WM condition, and an interaction between the two predictors. We found a non-significant effect of the standard deviation of the number of stimuli shown ($\beta_{sd\left(n\;stim\right)}$ = 0.007, s.e. = 0.004, *p* = 0.132; figure 3b) but a significant effect of the WM condition ($\beta_{WM}$ = 0.028, s.e. = 0.007, *p* < 0.001; figure 3b). No interaction was found between the two predictors ($\beta_{WM*sd\left(n\;stim\right)}$ = 0.007, s.e. = 0.007, *p* = 0.266; figure 3b). As a last control, we selected the trials with the most frequent set size on each condition—thus reducing variability to a minimum—and computed metacognition considering these trials only. We still found a difference favouring the WM condition using this subset of trials (*t*_100_ = 2.31, *p* = 0.023, *d* = 0.23; figure 3c) and this difference was sufficiently large to matter as the equivalence test was not significant (*t*_100_ = –0.96, *p* = 0.169; figure 3c). Overall, these results suggest that the variability in the number of items presented on each condition cannot account for the observed results.

We also explored whether the effect of the IM condition in confidence was different regarding correct and incorrect trials by adding an interaction term between the accuracy of the response and the IM condition predictors to our original regression analysis. Contrary to our first result, we found that confidence was higher in the IM condition ($\beta_{IM}$ = 0.034, s.e. = 0.017, *p* = 0.048), an effect that had a negative interaction with the accuracy of the response ($\beta_{IM*accuracy}$ = –0.22, s.e. = 0.023, *p* < 0.001). This suggests that confidence was differentially affected by the IM condition depending on whether the response was correct or incorrect, with a mean positive effect in incorrect trials that becomes negative in the correct ones. The rest of the predictors had similar values to the ones found in the pre-registered regression analysis ($\beta_{n\;stim}$ = –0.124, s.e. = 0.004, *p* < 0.001; $\beta_{accuracy}$ = 1.389, s.e. = 0.017, *p* < 0.001). However, when adding a random effects structure for all the predictors (see §3), the IM did not have a positive effect on confidence judgements (*t*‐test against zero for the distribution of beta values: *t*_100_ = –0.70, *p* = 0.488, *d* = 0.07). The interaction effect remained significant (*t*_100_ = –8.97, *p* < 0.001, *d* = 0.89). Overall, the results reveal that confidence levels were higher for incorrect trials compared with correct trials in the IM condition (controlling for accuracy and the number of stimuli presented on each trial), but it did not differ between IM and WM. Noteworthy, this latter model specification fitted the data substantially better (ΔAIC_model 2 – model 3_ = 602.55).

**Figure 4.**
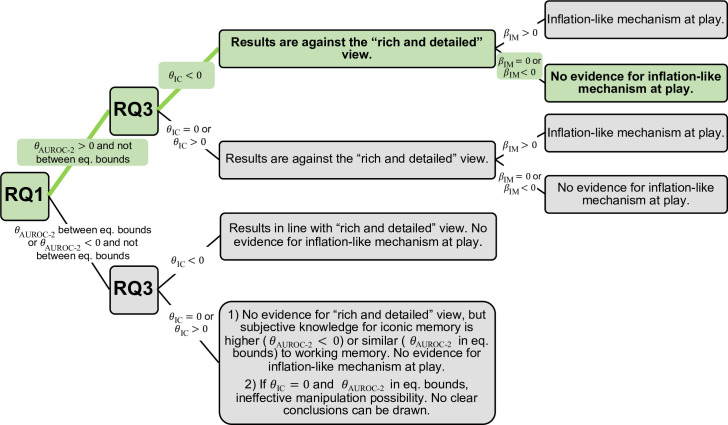
Diagram for interpretation of all the possible outcomes of the study. Highlighted in green are the results obtained.

For clarity we include (figure 4) an outcome interpretation diagram (similar to the pre-registered one), highlighting the pathway corresponding to the results empirically obtained.

## 5. Discussion

In the present study, we compared metacognitive sensitivity for IM and WM representations to inform the debate about the richness of perception. Our results suggest that while IM has a greater informational capacity—allowing participants to process a larger number of items—this information may not be as consciously accessible as the information available for WM representations. This is supported by the finding that greater metacognitive sensitivity levels were found in this kind of memory, challenging the idea that our conscious perception is rich and detailed.

Our results seem, at face value, opposed to previous results comparing metacognition in sensory (iconic and fragile) memory and WM. For instance, Vandenbroucke *et al*. [4] reported similar metacognitive sensitivity levels between these memories. However, their findings—specially for the IM and WM comparison—appears to be restricted to change detection tasks. When they employed an identification task—conceptually similar to our protocol—lower metacognitive sensitivity was found in the IM compared with WM. Our results replicate this pattern using a different set of stimuli. In this line, identification tasks seem better suited to address the question of how detailed our conscious perception is. Indeed, high performance in detection may be driven by a hunch that the stimuli have changed, there could be hints of motion, etc., whereas successful identification more strongly suggests that participants perceived the stimuli with at least some degree of detail ([12]; although high resolution perception may still not be guaranteed, see below). In addition, change detection tasks can induce differences in the decision criterion between memory conditions (as happened in [4]), which can also influence metacognitive sensitivity measures [27].

While our findings align with some previous research employing identification tasks, they appear to diverge from those of Sligte *et al*. [47]. In their task, participants were asked to detect a change between two sets of stimuli, and then to identify the ‘pre-change’ stimuli between a set of four stimuli. They found higher identification performance in IM representations compared with WM ones. However, it remains controversial whether high identification performance guarantees having seen the stimulus with high resolution, as indeed some degraded information from the pre-change item could support correct discrimination between the other three stimuli [12]. In this sense, our paradigm (and well as that of [4]) has the strength to appeal to metacognitive judgements instead of performance to evaluate the conscious content, which are known to have several advantages for this aim [25].

Previous research has shown that mixing different levels of difficulty in perceptual decision-making tasks can artificially inflate metacognition estimates [45]. This issue is relevant in our task as we employed two independent staircases for controlling performance, which could lead to a spurious difference in metacognitive levels if one condition ended up with more mixing of different difficulty levels (represented by the amount of items shown on each trial, or set size, in our task). Our exploratory control analyses suggest that indeed there was a difference in set size variability between the two memory conditions. However, we found that this variability is unlikely to account for our results for several reasons: (i) higher set size variability was found in the IM condition, which means that, if anything, it should lead to higher metacognitive levels for the IM condition; (ii) set size variability did not predict metacognitive levels; and (iii) computing metacognitive sensitivity using only trials with the most frequent number of items presented (i.e. only with one difficulty level) did not change the results. In summary, we believe that these control analyses further strengthen the results obtained.

Our results challenge the rich and detailed view. However, it remains to be explained why we feel that we have a rich conscious perception. A proposed explanation to solve this conundrum is the mentioned mechanism of inflation, defined as the overestimation of the quality of our perceptual inputs [12,15,18]. We expected to find such a mechanism expressed in higher confidence levels in the IM condition. However, we found the opposite pattern: overall lower levels of confidence in the IM condition. This suggests that an inflation-like phenomenon may not be at play in our task. However, inflation is most commonly observed in tasks that engage information processing mechanisms akin to those present in peripheral vision. For instance, by using experimental paradigms that were specially designed to mimic features of peripheral vision (crowding and summary statistics) Odegaard *et al*. [18] found that participants reported higher confidence in discrimination judgements and higher rate of false alarms on detection judgements. In the same line, Solovey *et al*. [20] found that participants adopt more liberal criteria for detecting stimuli at the periphery compared with central vision. While we argued that our task could in principle induce such a similar process—as for instance a higher number of items were presented in the IM condition leading to noisier percepts—we did not manipulate visual spatial processing directly and therefore the absence of evidence for such a mechanism should be taken cautiously.

Furthermore, our task required fine-grained identification of letters, which probably requires more involvement of foveal vision. This may help explain the overall lower confidence in the IM condition, given that the metacognitive system seems to track the capabilities of the perceptual system as suggested by Sharvashidze *et al*. [48]. Indeed, the authors found that task requirements critically modulate the presence or absence of metacognitive biases in the visual field, with overconfidence in the periphery arising in cases where peripheral vision is preferred (e.g. a scene categorization task as in the [48] study).

Interestingly, Odegaard *et al*. [18] found that, in the realm of confidence judgements, the inflation effect is present specifically on incorrect trials. Along these lines, when we included an interaction term between the memory condition and response accuracy we observed a positive effect of the IM condition on confidence, particularly in incorrect trials, which is consistent with these findings supporting the inflation account. Nevertheless, this result appeared to be sensitive to model specification: when including random effects structure for all the predictors, the main positive effect of the IM on confidence was not present. In other words, there were no differences between IM and WM confidence levels. Given that this second model achieved a better fit, the results suggest that we did not find evidence for inflation as stated in RQ2. Noteworthy, however, the interaction between the accuracy of the response and the IM condition remained significant in the second model. Whether higher confidence in incorrect trials compared with correct trials in the IM condition (controlling by accuracy and the number of items presented) constitutes evidence for inflation itself is debatable and it was not a pre-registered interpretation of this study. Overall, we believe that future research should further investigate the conditions under which inflation effects on confidence judgements emerge.

Despite the mentioned strengths of the employed paradigm, it does suffer from its own limitations. First, alternative explanations can account for the data. For instance, as the only difference between conditions was the duration of the retention interval, a higher metacognitive ability might be the result of longer reflection on the rich content of the iconic information, which can also be related to the higher levels of confidence found in the WM condition. Furthermore, this longer retention interval may also induce forgetting, resulting in a lower number of items recalled. Such an alternative explanation is possible in principle and, importantly, would not imply a challenge for the rich view. Further research could employ other forms of proving working and IM metacognition, such as employing pre- and post-stimuli cues [2,4], with the aim of distinguishing between possible mechanisms underlying our pattern of results. In this line, experiment 2 of the Vandenbroucke *et al*. [4] study may already provide evidence of a similar pattern of results (higher IM capacity but lower metacognitive sensitivity compared with WM) in an identification task that does not depend on the duration of the retention interval.

Second, as emphasized by Haun *et al*. [49], forced-choice paradigms like the one employed here may underestimate the richness of perception: even if participants cannot identify the specific alternative required by the task, they may still consciously experience broad categorical or feature-level information (e.g. ‘letter-like figures’), which our design does not capture. This concern has motivated the development of paradigms allowing for more comprehensive reports of visual experience [9,50], though these approaches come with their own challenges [51]. The fact that our experimental design may underestimate the richness of perception is an issue that our study does not solve itself. It should also be noted, however, that the proponents of the rich view have favoured the use of metacognitive measures to index consciousness [49]. Future research could aim to combine metacognitive measures in tasks that allow more comprehensive reports of visual experience to further inform the debate on the richness of the content of conscious perception.

Finally, although it is a rationale based on a previous study supporting the rich view [4], it can be argued that it is not a core claim made by this proposal that IM metacognition has to be alike to the WM one. Furthermore, based on the assumption that metacognitive sensitivity itself indexes consciousness [25], our results showed above-chance metacognitive sensitivity for IM information, meaning that subjects could consciously access at least some of the information of IM. Overall, while our results challenge the idea that IM provides the same level of metacognitive access as WM, they do not rule out the possibility that IM supports some degree of conscious experience that is accessible by metacognitive monitoring.

Our results also speak to the broader debate on the relationship between phenomenal and access consciousness. Block [52,53] distinguished phenomenal consciousness (the qualitative, ‘what-it-is-like’ aspect of experience) from access consciousness, which involves the availability of information for reasoning, report or action. Proponents of the rich view often appeal to this distinction, suggesting that phenomenal consciousness may overflow access, such that more is consciously experienced than can be reported or accessed for decision-making, illustrated, for instance, by classic experiments proving IM, as mentioned. One can speculate that some of this phenomenological part of the experience was captured by metacognitive introspection given that, despite being lower than in the WM condition, the metacognitive ability of the participants in the IM condition was still above chance (as noted above). Another, not mutually exclusive, point related to Block’s ideas is that one can argue that phenomenal consciousness can also overflow ‘metacognitive’ access and that metacognitive measures cannot detect all the rich conscious content of the IM. Metacognitive inefficiency, therefore, reflects unavailability for metacognition, not unconsciousness [25]. This may explain the lower metacognitive sensitivity levels found in the IM condition. Despite this being a possible scenario, Michel [25] argues that: (i) this problem applies to virtually all metrics of consciousness; (ii) it is not reasonable to expect that subjects are ‘metacognitively blind,’ since many of our social and scientific practices presuppose the reliability of subjective reports. Indeed, proponents of the rich view have also endorsed metacognitive measures, as discussed above [4,49].

Finally, our findings, at least theoretically, seem to be in line with higher order theories [54], which propose two stages to account for conscious perception: a first-stage process that drives task performance which is not itself conscious, and a second-stage process that monitors changes in the first stage to determine what enters conscious awareness. Indeed, if one accepts the validity of metacognitive measures of consciousness [25], the reduction of metacognitive sensitivity in IM may reflect the absence of the extra information presented in this memory at the second-stage level, which supports metacognition. This may help to explain why, for instance, subjects do not detect unexpected items placed in the uncued locations in Sperling-like paradigms [55]. Lau & Rosenthal [54] argue for the case of inflation in such cases; however, as previously detailed, we could not find strong evidence favouring this view in our data.

Overall, our findings suggest that the higher capacity of IM in identification tasks seems to be based on information that is not as consciously accessible as the WM one. While naturally our results do not settle the debate completely, this challenges previous interpretations of similar experimental paradigms that favoured the rich and detailed view of conscious perception.

## Data Availability

All data, materials and analyses scripts can be found at: https://osf.io/nc5hw/. The stage 1 pre-registration can be found here [56]. Supplementary material is available online [57].
